# Supplementary material for: HOTAIR Promotes the Hyperactivation of PI3K/Akt and Wnt/β-Catenin Signaling Pathways via PTEN Hypermethylation in Cervical Cancer
Source: Cells. 2024 Sep 4;13(17):1484. doi: 10.3390/cells13171484 (PMC11394386; doi:10.3390/cells13171484)
Supplement: Supplementary file 1 [file cells-13-01484-s001.zip › Supplementary Figures.pdf]

# Supplementary Figures

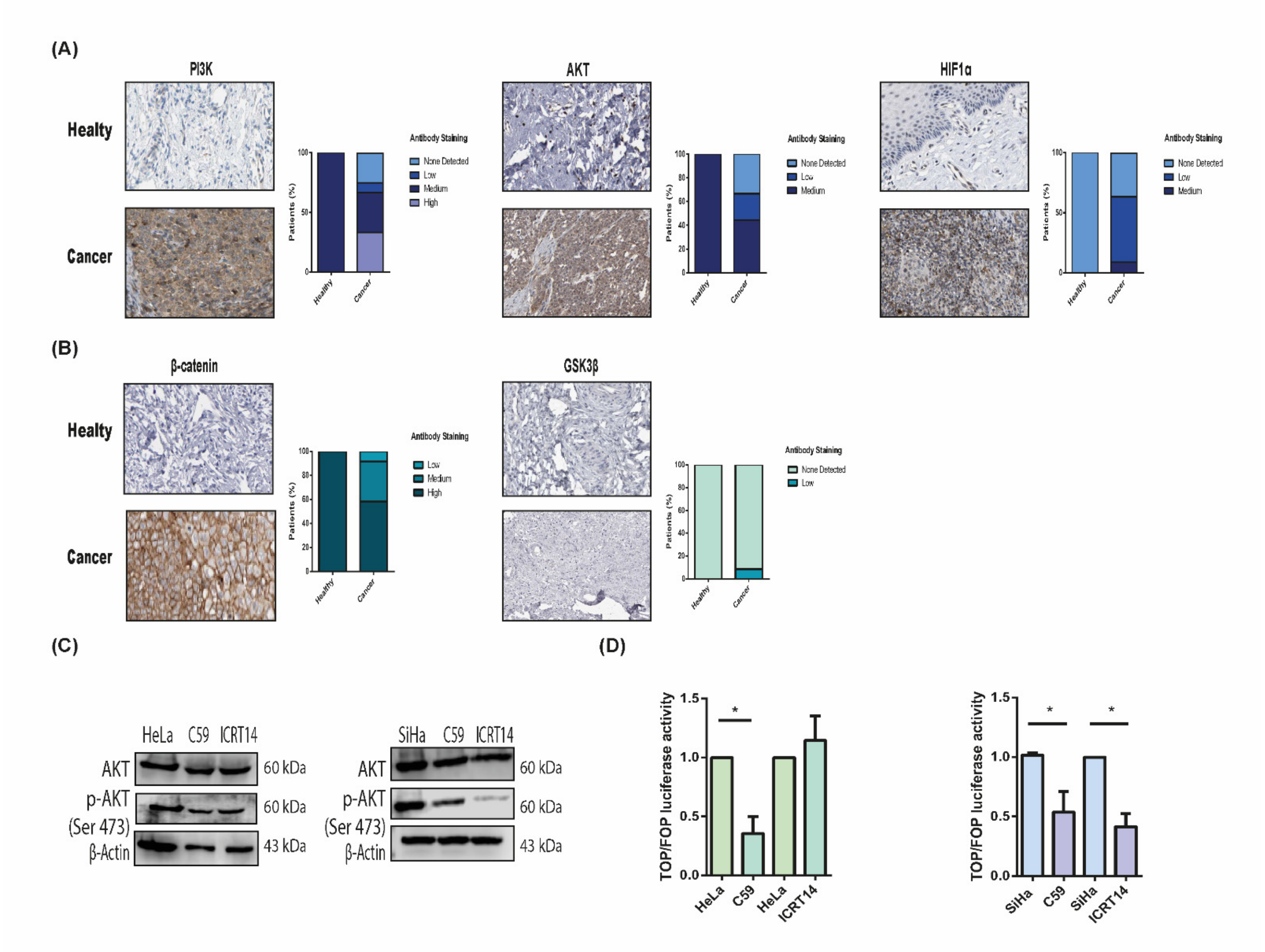


**Supplementary Figure S1. Expression of key components of PI3K/AKT and Wnt/ β-catenin in Cervical Cancer and normal tissues. (A-B)** Protein atlas *In silico* meta-analysis to PI3K/AKT and Wnt/β-catenin signaling pathways efectors in CC (13) in comparison to healthy samples (3). (**C**) Western blots to PI3K/AKT pathway treated with C59 and ICRT14 Wnt/β-catenin inhibitors in CC cell lines. (**D**) TOP Flash assay for C59 and ICRT14 inhibitors in CC cell lines. $p < 0.05$ (*).

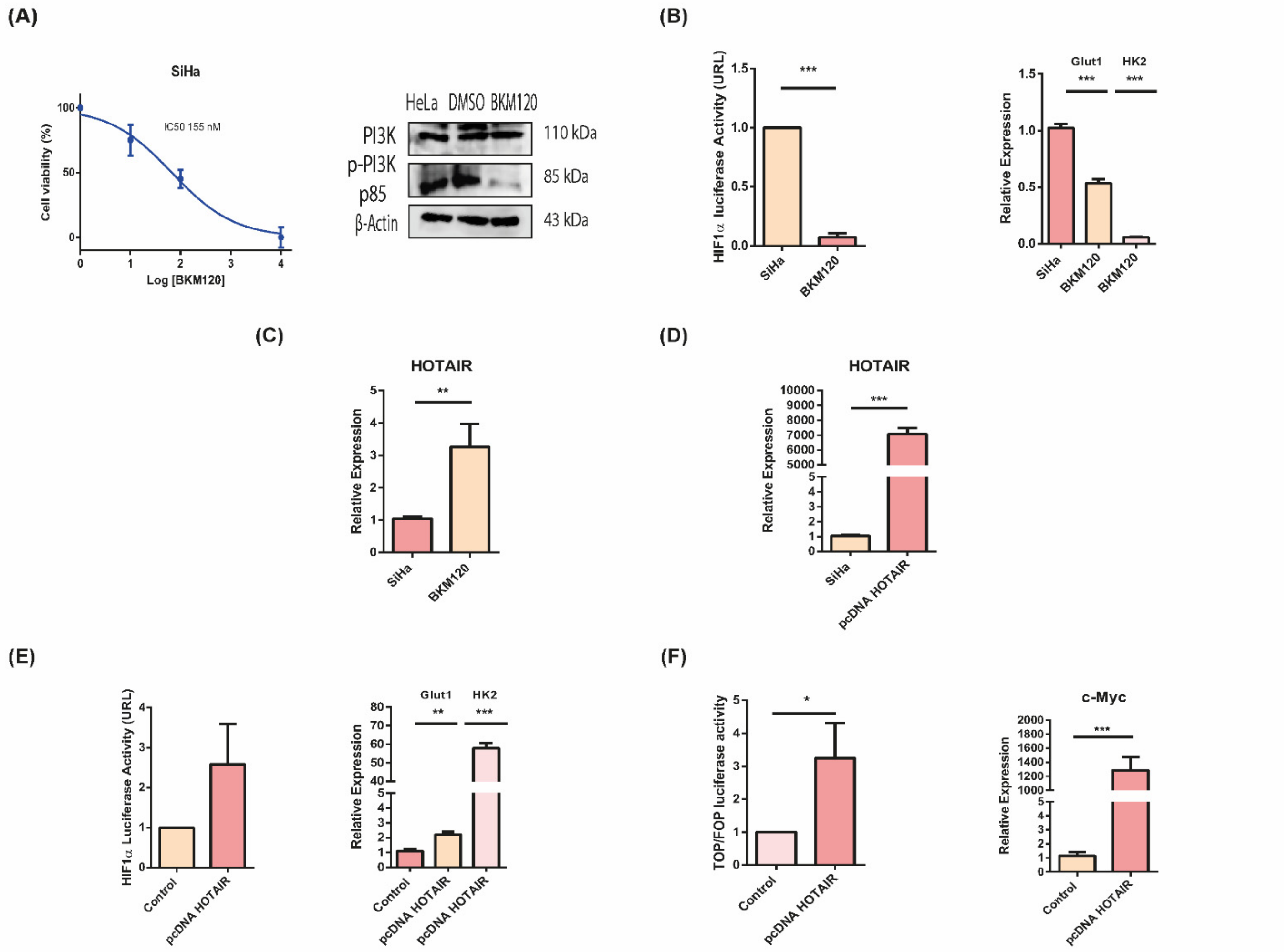


**Supplementary Figure S2. PI3K/AKT regulates HOTAIR expression and Wnt/β-catenin activation in SiHa cell line.** (**A**) IC50 and Western Blot of PI3K/AKT pathway with BKM120 inhibitor. (**B**) BKM120 inhibits transcriptional activity of HIF1*α* by measuring luciferase reporter and Glut1 and HK2 expression in SiHa cells. (**C**) HOTAIR expression with BKM120 inhibitor in SiHa cell line. (**D**) HOTAIR overexpression in SiHa cell line. (**E**) HOTAIR overexpression increases HIF1*α* transcriptional activity by luciferase activity and HIF1*α* targets expression. (**F**) HOTAIR overexpression increases Wnt/β-catenin transcriptional activity evaluated by TOP Flash activity and c-Myc expression. $p < 0.05$ (*). $p < 0.01$ (**). $p < 0.001$ (***).

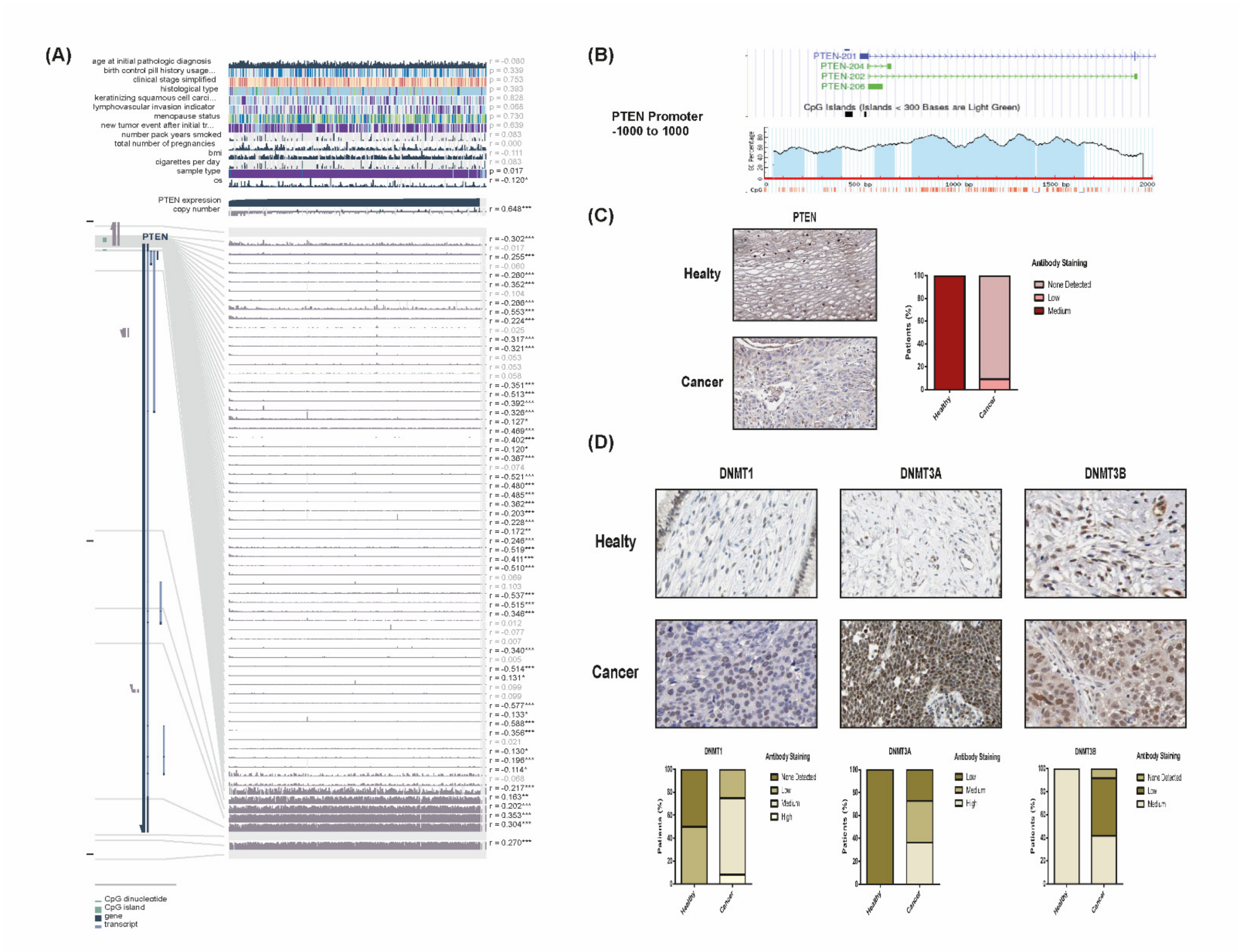


**Supplementary Figure S3. PTEN promoter is methylated in Cervical Cancer. (A-B)** In silico analysis to PTEN methylation patron on 317 CC samples and sequence analysis through MEXPRESS data base and Genome browser-MethPrimer respectively. (**C-D**) Protein atlas In silico meta-analysis to PTEN and DNMT1, 3A and 3B in CC (13) in comparison to healthy samples (3).
